# Risk factor studies of age-at-onset in a sample ascertained for Parkinson disease affected sibling pairs: a cautionary tale

**DOI:** 10.1186/1742-7622-4-1

**Published:** 2007-04-04

**Authors:** Jemma B Wilk, Timothy L Lash

**Affiliations:** 1 Boston University School of Medicine, Boston, MA, USA; 2 Boston University School of Public Health, Boston, MA, USA

## Abstract

An association between exposure to a risk factor and age-at-onset of disease may reflect an effect on the rate of disease occurrence or an acceleration of the disease process. The difference in age-at-onset arising from case-only studies, however, may also reflect secular trends in the prevalence of exposure to the risk factor. Comparisons of age-at-onset associated with risk factors are commonly performed in case series enrolled for genetic linkage analysis of late onset diseases. We describe how the results of age-at-onset studies of environmental risk factors reflect the underlying structure of the source population, rather than an association with age-at-onset, by contrasting the effects of coffee drinking and cigarette smoking on Parkinson disease age-at-onset with the effects on age-at-enrollment in a population based study sample. Despite earlier evidence to suggest a protective association of coffee drinking and cigarette smoking with Parkinson disease risk, the age-at-onset results are comparable to the patterns observed in the population sample, and thus a causal inference from the age-at-onset effect may not be justified. Protective effects of multivitamin use on PD age-at-onset are also shown to be subject to a bias from the relationship between age and multivitamin initiation. Case-only studies of age-at-onset must be performed with an appreciation for the association between risk factors and age and ageing in the source population.

## Background

Studies of the relation between a risk factor and age-at-onset of a disease, as opposed to the relation between a risk factor and the occurrence of disease, are designed to investigate the hypothesis that the risk factor causes the disease to occur earlier than it would have occurred without exposure to the risk factor. For example, the risk factor may accelerate the disease process, so that the average disease-free survival time from birth to disease onset is shorter for those exposed to the risk factor than for those without exposure to the risk factor. Conventional epidemiologic designs can investigate these hypotheses, but when only cases are available, age-at-onset studies are a convenient way to investigate associations between risk factors and disease. The limitation of the case-only design is that the analysis cannot account for potential age differences between those exposed and unexposed to the risk factor, which would suggest a difference in the onset of exposed and unexposed cases, but without any etiologic relevance.

Though many epidemiologists appreciate this inherent shortcoming of using age-at-onset of disease as an outcome, it has become an increasingly common outcome measure in genetic epidemiology studies of late onset diseases such as Parkinson and Alzheimer disease [1,2]. Genetic studies that were designed for linkage analysis of a disease often enroll only cases, so these case-only studies can investigate the association between risk factors and age-at-onset, but not the association between risk factors and the occurrence of disease itself. While such studies of age-at-onset may be appealing, since identifying factors related to delayed onset might facilitate the development of therapeutic agents or public health interventions, a study associating age-at-onset with a risk factor that may vary over time is susceptible to confounding by age itself. Age may not only be related to age-at-onset but also be related to the prevalence of exposure to the risk factor. Since only cases appear in the analysis, an underlying association between the risk factor and age in the source population that gave rise to the cases can be too easily ignored [3], and a difference in age-at-onset of disease could be interpreted to have etiologic importance.

The comparison of age-at-death for left-handers with age-at-death for right-handers provides a classic illustration [4,5]. The authors observed a 9 year earlier age-at-death for left-handers [5], and speculated that it may have arisen from prenatal or perinatal birth stressors, genetic effects and intrauterine hormones that reduced immune function, or accidents in an environment designed for a right-handed majority [4]. They even noted that deaths by accidents were more common among left-handers than right-handers. In reality, many left-handers born in the early part of the twentieth century were forced to use their right hand, a practice that has disappeared over time. Those born later in the century were less likely to be forced to use their right hand. In these studies conducted toward the end of the twentieth century, persons dying at old age were less likely to be classified as left-handed because of a birth cohort effect [6]. Those dying at young ages, but at the same calendar time, were not as likely to have suffered this social pressure, so were correctly classified as left handed. Deaths due to accidents were more common among left-handers because left-handers were younger and deaths among young people are more likely accidental. Birth cohort effects best explain the difference in age-at-death, which could have been revealed had denominator data, such as mortality rates, been available. Subsequent prospective cohort studies have shown that left-handers and right-handers have equivalent mortality rates [7-9]. These studies of age-at-death were difficult to interpret because secular trends influenced the prevalence of left-handedness, which resulted in an underlying association between left-handedness and age in the source population. We illustrate similar concerns about studying age-at-onset in case-only studies and provide a cautionary tale from a research study of age-at-onset of Parkinson's Disease.

Parkinson's disease (PD [MIM 168600]) is a neurodegenerative disease with onset in mid to late life, and it is usually characterized by tremor, rigidity, bradykinesia, and therapeutic response to levodopa. PD is caused by the slow degeneration of dopamine-producing neurons in the substantia nigra, but the etiology of this degeneration is not well understood [10]. To date, several genome wide scans for PD have been performed and multiple chromosomal regions have been implicated in PD etiology [11-13], though the population attributable fraction for each individual gene or region appears to be low. Epidemiologic studies of non-genetic factors have suggested that a history of head trauma may increase PD risk [14], while coffee drinking and cigarette smoking may decrease PD risk. In a meta-analysis, Hernan and colleagues reported a relative risk (RR) for PD of 0.69 [95% confidence interval (CI) 0.59–0.80) in coffee drinkers compared with non-coffee drinkers and a RR of 0.59 (95% CI 0.54–0.63) for ever smokers compared to never smokers [15].

The observation of decreased risk for PD in coffee drinkers and cigarette smokers fostered the hypothesis that substances in coffee and cigarettes, namely caffeine and nicotine, protect against the neurodegenerative mechanisms that ultimately lead to PD. The potential neuroprotective effect of caffeine is supported by findings from mouse models of PD, in which a low dose of caffeine before administration of the neurotoxin MPTP attenuated dopamine depletion [16]. Similar neuroprotective effects for nicotine have been observed in animal models [17].

A recent report extended the hypothesis that smoking protects against PD occurrence to the hypothesis that smoking delays onset among cases [18]. They compared average age-at-onset in ever-smokers with never-smokers, finding that onset age was significantly older among ever-smokers compared to never-smokers (a difference in onset symptoms of 3.5 years with 95% CI from 1.6 to 5.4), and argued that a bias explaining these results was unlikely. Our studies suggest that the validity of this inference, on the basis of an association between smoking and age-at-onset in a case-only design, is questionable. In this report, we used data from the *Gene*PD study to evaluate whether the associations between coffee drinking and cigarette smoking and age-at-onset of PD reflected secular trends in the prevalence of exposure to these risk factors. We re-evaluated the prior reports of an association between head trauma and multivitamin use with age-at-onset in this sample. Previous reports have reviewed the pitfalls of case-only designs to assess gene-environment interactions [19,20]; we focus on the subset of case-only analyses that assess age-at-onset, some of which make use of study populations originally enrolled to investigate genetic hypotheses.

## Analysis

The *Gene*PD study is a multi-center study of affected relative pairs with PD that was designed for genetic linkage analyses [11,21]. Recruitment began in 1997 and is ongoing. Participants in the current analysis were born between 1901 and 1963 and diagnosed with PD between 1965 and 2002. Recruitment included both prevalent and incident cases, with a median difference between age-at-onset and enrollment of 7.5 years (range: 0 to 40 years). Risk factor data on history of head trauma and consumption of coffee, cigarettes, and multivitamins were collected by questionnaire and specifically inquired about the year(s) of exposure. Participants were classified as exposed to a risk factor only when exposure occurred before the age-at-onset. The case-only design of this familial study lends itself to studies that relate age-at-onset of PD to risk factor exposures. The *Gene*PD study previously reported an association between a history of head trauma and younger age of PD onset, and associations between the use of multivitamins and greater pack-years of smoking with older age-at-onset [21]. At the time of this earlier publication, coffee drinking was not strongly associated with age-at-onset of PD. As recruitment has continued, these associations have continued to be evaluated in the growing sample.

The findings previously reported for multivitamin use and pack-years of smoking associated with later age-at-onset of PD are examples of the difficulty of studying associations between non-genetic risk factors and age-at-onset. While the number of pack-years is a commonly used measure of smoking dose and duration, its use to evaluate an association between smoking and onset age is inherently biased by the role of age in both the dependent and independent variable. The finding of later age-at-onset with larger number of pack-years of smoking reflects that older smokers have smoked for a longer time than younger smokers and also is likely to reflect a birth cohort effect influencing the age of smoking initiation. The finding should not be interpreted as suggesting that smoking delays the onset of PD, the protective association reported in the meta-analysis notwithstanding [15]. Although it is plausible that smoking both reduces the risk of PD occurrence and delays its onset, the association between pack-years of smoking and age-at-onset of PD should not be relied upon to reach either conclusion.

The reported association between multivitamin use and older age-at-onset was similarly plagued by the association between multivitamin use and age in the source population. We evaluated the association between multivitamin use and PD age-at-onset by calculating the average age-at-onset of PD in a reference group of never-users of multivitamins and comparing it with the average age-at-onset in multivitamin users in which we sequentially incorporated cases who began using multivitamins at ever increasing ages. We observed that the association of a later age of PD onset with multivitamin use appeared only when we included users who initiated multivitamin use at age 70 years or older. In fact, restricting the users to those who initiated multivitamin use at the youngest ages showed an association with earlier onset age. This difference likely reflects a birth cohort effect because younger cases, born in more recent health conscious years, were more likely to use multivitamins before onset of PD compared to the group of cases who were never users of multivitamins. Table 1 reports the estimates of the mean differences in age-at-onset and their 95% confidence intervals from a linear regression model relating multivitamin use to PD age-at-onset across the initiation age cutoffs. The inconsistency in the direction and magnitude of the difference across exposure age cutoffs does not suggest a true effect. If multivitamin use was truly protective, we would expect consistency in the direction of the effect across exposure age cut-offs, as is the case in the head trauma example below. Rather, it suggests that the original finding with a dichotomous multivitamin use variable was biased by the ever-increasing proportion of the source population taking vitamins as they age.

**Table 1:** The association of multivitamin use and head trauma with PD age-at-onset.

	Multivitamin use			Head trauma		
Exposure Age	N exposed	Difference in age-at-onset (years)	95% CI	N exposed	Difference in age-at-onset (years)	95% CI
≤30	62	-2.0	-5.2, 1.2	67	-4.9	-7.7, -2.0
≤40	92	-1.9	-4.5, 0.8	73	-5.1	-7.8, -2.5
≤50	128	0.3	-2.1, 2.7	79	-4.8	-7.3, -2.3
≤60	163	1.2	-0.9, 3.3	88	-4.2	-6.6, -1.9
≤70	187	2.6	0.6, 4.6	90	-4.0	-6.4, -1.6
≤80	194	3.1	1.1, 5.2	91	-3.8	-6.2, -1.5

Analysis performed by sequentially increasing the upper age limit for exposure, but maintaining a constant reference group of never-users of multivitamins (N = 342; mean age-at-onset 60.6 years) or no history of head trauma (N = 451; mean age-at-onset 62.4 years). Negative β estimates reflect a younger mean age-at-onset in the exposed group.

As a contrast with the result for multivitamin use, we present a parallel analysis of the relation between head trauma and PD onset age (Table 1). A history of head trauma was present when participants reported a head injury severe enough to cause a loss of consciousness, blurred/double vision, dizziness, vertigo, seizures, convulsions, temporary memory loss, or paralysis. The reported association between a history of head trauma and younger age-at-onset of PD appears to be robust to the bias observed in the analysis of multivitamin use. The finding is plausible because of the established association between head trauma and increased risk of PD, but the result associating head trauma with younger onset of PD may still be plagued by the biases of studying age-at-onset in prevalent cases and the potential for differential recall bias due to age-related memory loss or survival with PD.

Given that the protective associations between coffee drinking and cigarette smoking and risk of PD have been replicated in many studies and in animal models, we have continued to investigate the association between these factors and age-at-onset of PD. To further evaluate our findings of an association between coffee drinking, smoking, and PD age-at-onset, we compared them to the associations between coffee drinking, smoking, and age-at-enrollment in the unselected sample of the population-based National Heart, Lung, and Blood Institute (NHLBI) Family Heart Study (FHS). FHS participants were recruited from existing family-based epidemiologic studies by one of two recruitment mechanisms 1) a random selection of 2000 participants yielding 588 families with complete clinic exams, or 2) a selection of 2000 participants with a reported family history of coronary heart disease yielding 657 families with clinic exams [22]. For comparison with the PD sample, only members of families recruited through random selection were studied.

We investigated the age-at-event associations in the *Gene*PD Study and FHS in parallel. Enrollment in FHS serves as a proxy event to compare to age-at-onset that is not expected to be associated with any exposure in a population sample. Since both are family designs, we used a generalized estimating equation to calculate the standard error of the beta estimates, which accounts for the multiple observations within a family. Age-at-onset of PD and age-at-enrollment in FHS were used as the quantitative dependent variables and independent variables for coffee drinking (yes/no), former smoking (yes/no), and current smoking (yes/no) at the time of PD onset or FHS enrollment were included in the model. For the study in *Gene*PD, the head trauma covariate (yes/no) was also included. For comparability, we restricted FHS subjects to those enrolled within the age range of PD subjects' ages-at-onset (20–88 years). The analyses were repeated restricting the individuals to age-at-onset or enrollment of at least 45 years. Results are reported in Table 2. Coffee drinking and current cigarette smoking are predictors of PD age-at-onset and age at-enrollment in both the full and age-restricted samples. The direction and estimate of the effect size between the *Gene*PD Study and the FHS sample are comparable, with substantially overlapping 95% confidence intervals.

**Table 2:** The association of coffee drinking and cigarette smoking with PD age-at-onset and with FHS age-at-enrollment.

	*Gene*PD – difference in age-at-onset of PD (years)		FHS – difference in age-at-enrollment (years)	
	β estimate*	95%CI	β estimate*	95%CI
All subjects	N = 542		N = 2017	
Coffee drinking	4.0	1.3, 6.7	3.0	1.6, 4.5
Former smoking	0.7	-1.3, 2.6	3.1	1.6, 4.6
Current smoking	-6.4	-9.9, -2.9	-5.0	-7.2, -2.9

Subjects ≥ 45 yrs	N = 496		N = 1380	
Coffee drinking	2.1	0.0, 4.1	1.3	0.3, 2.4
Former smoking	0.6	-1.1, 2.2	0.3	-0.7, 1.3
Current smoking	-4.2	-7.4, -1.1	-2.9	-4.5, -1.3

*Positive β estimates reflect an older mean age-at-onset (or enrollment) in the exposed group.

## Conclusion

The results presented here cast considerable doubt that a valid relation exists between multivitamin use, coffee drinking, or cigarette smoking and age-at-onset of PD. If multivitamin use truly delayed the onset of PD, we would expect to observe the largest difference in age-at-onset among the group that initiated their multivitamin use at the youngest ages, but this pattern was not observed. For coffee drinking, it is tempting to see the results within the *Gene*PD study in conjunction with prior studies of coffee and PD risk and speculate that coffee has potential neuroprotective effects that delay onset of PD. However, the comparison of the *Gene*PD association with the FHS association suggests that the finding is related to a secular trend in coffee consumption, with older individuals being more likely to self identify as regular coffee drinkers than younger individuals. The result for current smoking does not support the hypothesis based on prior studies of PD risk, but exemplifies a population trend for smoking cessation at older ages. These findings for PD age-at-onset should not be interpreted as a refutation of prior findings associating these exposures with PD risk.

One might ask whether it is ever possible to perform an unbiased study of the association between risk factors and age-at-onset? We believe that the answer is yes, but that it requires thoughtful study design and knowledge about how the risk factors are related to age and ageing in the source population [23]. It is unlikely that we can validly study risk factors that are inherently related to age as simple categorical indicators. The example of multivitamin use and PD age-at-onset demonstrates this difficulty, in which a protective association was reported based on a small number of relatively older onset PD individuals who initiated multivitamin use just before diagnosis, but not necessarily at a time with a relevant induction period. Their initiation of multivitamin use may have been related to self-medication of preclinical symptoms of PD.

Valid studies of age-at-onset require no underlying association between the risk factor and aging or birth cohort in the source population. They must also consider whether a sufficient induction time has passed for the risk factor to have an effect. When these criteria and others [23] cannot be satisfied, age-specific or standardized risks or rates, or a population-based case-control design, must be used to study the association between the risk factor and outcome. These designs allow the investigator to disaggregate the relation between aging and the prevalence of the risk factor, using familiar methods to control confounding in the design or analysis. When prior knowledge strongly suggests that the prevalence of the risk factor changes with age in the source population, case-only studies may support a relation between the risk factor and age-at-onset, regardless of whether the inference is justified.

## Competing interests

JBW receives salary support from the *Gene*PD study and is actively involved in studies of genetic factors influencing age-at-onset of PD. TLL receives consulting fees from attorneys representing clients involved in litigation regarding PD etiology.

## Authors' contributions

JBW conceived of the study design to contrast results for PD age-at-onset with age-at-enrollment in the FHS population and drafted the manuscript and tables. TLL drafted the review of the handedness literature and contributed to the interpretation of the results. Both authors participated in revision and approved the final work.

## References

[B1] Li YJ, Oliveira SA, Xu P, Martin ER, Stenger JE, Scherzer CR, Hauser MA, Scott WK, Small GW, Nance MA, Watts RL, Hubble JP, Koller WC, Pahwa R, Stern MB, Hiner BC, Jankovic J, Goetz CG, Mastaglia F, Middleton LT, Roses AD, Saunders AM, Schmechel DE, Gullans SR, Haines JL, Gilbert JR, Vance JM, Pericak-Vance MA, Hulette C, Welsh-Bohmer KA (2003). Glutathione S-transferase omega-1 modifies age-at-onset of Alzheimer disease and Parkinson disease. Hum Mol Genet.

[B2] Blomqvist ME, Silburn PA, Buchanan DD, Andreasen N, Blennow K, Pedersen NL, Brookes AJ, Mellick GD, Prince JA (2004). Sequence variation in the proximity of IDE may impact age at onset of both Parkinson disease and Alzheimer disease. Neurogenetics.

[B3] Rothman KJ (2002). Introduction to Epidemiologic Thinking. Epidemiology: An Introduction.

[B4] Halpern DF, Coren S (1988). Do right-handers live longer?. Nature.

[B5] Halpern DF, Coren S (1991). Handedness and life span. New Engl J Med.

[B6] Rothman KJ (1991). Left-handedness and life expectancy (letter). N Engl J Med.

[B7] Wolf PA, D'Agostino RB, Cobb J (1991). Left-handedness and life expectancy (letter). N Engl J Med.

[B8] Salive ME, Guralnik JM, Glynn RJ (1993). Left-handedness and mortality. Am J Public Health.

[B9] Basso O, Olsen J, Holm N, Skytthe A, Vaupel JW, Christensen K (2000). Handedness and mortality: A follow-up study of Danish twins born between 1900 and 1910. Epidemiology.

[B10] Checkoway H, Nelson LM (1999). Epidemiologic approaches to the study of Parkinson's disease etiology. Epidemiology.

[B11] DeStefano AL, Golbe LI, Mark MH, Lazzarini AM, Maher NE, Saint-Hilaire M, Feldman RG, Guttman M, Watts RL, Suchowersky O, Lafontaine AL, Labelle N, Lew MF, Waters CH, Growdon JH, Singer C, Currie LJ, Wooten GF, Vieregge P, Pramstaller PP, Klein C, Hubble JP, Stacy M, Montgomery E, MacDonald ME, Gusella JF, Myers RH (2001). Genome-wide scan for Parkinson's disease: the GenePD Study. Neurology.

[B12] Scott WK, Nance MA, Watts RL, Hubble JP, Koller WC, Lyons K, Pahwa R, Stern MB, Colcher A, Hiner BC, Jankovic J, Ondo WG, Allen FH, Goetz CG, Small GW, Masterman D, Mastaglia F, Laing NG, Stajich JM, Slotterbeck B, Booze MW, Ribble RC, Rampersaud E, West SG, Gibson RA, Middleton LT, Roses AD, Haines JL, Scott BL, Vance JM, Pericak-Vance MA (2001). Complete genomic screen in Parkinson disease: evidence for multiple genes. JAMA.

[B13] Pankratz N, Uniacke SK, Halter CA, Rudolph A, Shults CW, Conneally PM, Foroud T, Nichols WC, Parkinson Study Group (2004). Genes influencing Parkinson disease onset: replication of PARK3 and identification of novel loci. Neurology.

[B14] Taylor CA, Saint-Hilaire MH, Cupples LA, Thomas CA, Burchard AE, Feldman RG, Myers RH (1999). Environmental, Medical, and Family History Risk Factors for Parkinson's Disease: A New England-Based Case Control Study. Am J Med Genet.

[B15] Hernan MA, Takkouche B, Caamano-Isorna F, Gestal-Otero JJ (2002). A meta-analysis of coffee drinking, cigarette smoking, and the risk of Parkinson's Disease. Ann Neurol.

[B16] Chen J-F, Xu K, Petzer JP, Staal R, Xu Y-H, Beilstein M, Sonsalla PK, Castagnoli K, Castagnoli N, Schwarzschild MA (2001). Neuroprotection by caffeine and A_(2A) _adenosine receptor inactivation in a model of Parkinson's Disease. J Neurosci.

[B17] Parain K, Hapdey C, Rousselet E, Marchand V, Dumery B, Hirsch EC (2003). Cigarette smoke and nicotine protect dopaminergic neurons against the 1-methly-4-phenyl-1,2,3,6-tetrahydropyriding Parkinsonian toxin. Brain Res.

[B18] De Reuck J, De Weweire M, Van Maele G, Santens P (2005). Comparison of age of onset and development of motor complications between smokers and non-smokers in Parkinson's disease. J Neurol Sci.

[B19] Albert PS, Ratnasinghe D, Tangrea J, Wacholder S (2001). Limitations of the case-only design for identifying gene-environment interactions. Am J Epidemiol.

[B20] Gatto NM, Campbell UB, Rundle AG, Ahsan H (2004). Further development of the case-only design for assessing gene-environment interaction: evaluation of and adjustment for bias. Int J Epidemiol.

[B21] Maher NE, Golbe LI, Lazzarini AM, Mark MH, Currie LJ, Wooten GF, Saint-Hilaire M, Wilk JB, Volcjak J, Maher JE, Feldman RG, Guttman M, Lew M, Schuman S, Suchowersky O, Lafontaine AL, Labelle N, Vieregge P, Pramstaller PP, Klein C, Hubble J, Reider C, Growdon J, Watts R, Montgomery E, Baker K, Singer C, Stacy M, Myers RH (2002). Epidemiologic Study of 203 Sibling Pairs with Parkinson's Disease: The *Gene *PD Study. Neurology.

[B22] Higgins M, Province M, Heiss G, Eckfeldt J, Ellison RC, Folsom AR, Rao DC, Sprafka JM, Williams R (1996). NHLBI Family Heart Study: Objectives and Design. Am J Epidemiol.

[B23] Boshuizen HC, Greenland S (1997). Average Age at First Occurrence as an Alternative Occurrence Parameter in Epidemiology. Int J Epidemiol.

